# Life History of the Invasive Big-Headed Ant *Pheidole megacephala* (Hymenoptera: Formicidae)

**DOI:** 10.3390/insects17100992

**Published:** 2026-09-25

**Authors:** Zhengying Yuan, Ning Nong, Zhifang Qin, Qionghua Gao

**Affiliations:** Guangxi Key Laboratory of Agro-Environment and Agric-Products Safety, College of Agriculture, Guangxi University, Nanning 530004, China

**Keywords:** *Pheidole megacephala*, big-headed ant, life history, caste differentiation, larval instars, larval setae

## Abstract

The African big-headed ant, *Pheidole megacephala* (Fabricius, 1793), ranks among the world’s 100 worst invasive alien species, yet its basic life history remains poorly documented. We combined laboratory rearing and field observations in subtropical China with light and scanning electron microscopy to characterize the morphology, development, caste differentiation and reproductive behavior of this ant across its life cycle. Larvae passed through three instars. Head capsule width separated the three instars, whereas setal morphology was both instar- and caste-specific, because worker- and soldier-destined third instars remained densely setose while reproductive larvae shed their setae and were nearly glabrous as prepupae, an external marker of caste fate before pupation. At 28 °C, non-reproductives developed from egg to adult in 28.73 ± 2.83 d with marked asynchrony, whereas reproductives shared the early schedule but required longer prepupal and pupal periods, totaling 37.60 ± 3.20 d. Caste production was seasonal, colonies were polygynous, and oviposition proceeded in discrete batches. Nests were cryptic and associated with moist, sheltered microhabitats. This first integrated life-history account identifies seasonal and microhabitat vulnerabilities that surveillance and control can exploit.

## Simple Summary

The big-headed ant is one of the most damaging invasive species worldwide, yet surprisingly little is known about how it develops, reproduces and nests. We reared colonies of this ant in the laboratory and inspected wild nests in subtropical China to fill this gap. We found that the larvae pass through three stages, and that the tiny hairs covering the larvae reveal not only the stage but also whether a larva will become a worker, a soldier or a future reproductive, long before this is otherwise visible. Workers and soldiers developed from egg to adult in about four weeks, whereas future reproductives needed about nine days longer. Colonies contained several egg-laying queens, and queens laid eggs in batches, so that new workers emerged continuously. In the field, colonies produced only workers in winter and early spring, and reared soldiers and reproductives mainly from late spring to autumn. Nests were hidden in moist, sheltered places such as rotting wood and leaf litter and were difficult to detect. These findings help explain why this ant spreads so successfully and identify when and where surveillance and control will work best.

## 1. Introduction

The African big-headed ant, *Pheidole megacephala* (Fabricius, 1793), ranks among the most damaging invaders of tropical and subtropical ecosystems and is listed by the IUCN as one of the 100 worst invasive alien species worldwide [1]. Native to sub-Saharan Africa, the species has been carried by human commerce to virtually all warm regions of the globe [2,3,4]. The species has been transported by humans for centuries, and its first documented occurrence outside Africa dates to the late eighteenth century, when it was described from Mauritius in 1793 [3]. Its introduced range now spans North, Central and South America and the Caribbean, southern Europe, southern and southeastern Asia, Australia and islands across the Pacific and Indian Oceans [2,3], including southern China, where established populations occur in Fujian, Guangdong, Guangxi, Hainan, Taiwan and Hong Kong [4,5].

Where it invades, *P. megacephala* displaces native ants and other ground-dwelling arthropods [6,7], and its disruption of a defensive ant–plant mutualism in East African savannas has been shown to cascade upward to alter predator–prey interactions between lions and their prey [8]. In agroecosystems, the ant protects honeydew-producing hemipterans and thereby aggravates crop damage [9,10], while in urban settings it infests houses, greenhouses and hospital premises [4,11] and has even been implicated in localized scalp hair shedding, reported as a newly recognized type of hair loss that mimics alopecia areata [12]. Although the species is an effective predator that can suppress crop pests such as sugarcane stem borers, coffee twig borers and subterranean termites [13,14,15], its documented costs to invaded ecosystems far outweigh any prospective value as a biological control agent.

Its invasion success is thought to rest on several features of its social organization. Colonies are polygynous and frequently unicolonial, workers showing no aggression towards conspecifics from nests hundreds or even thousands of kilometers apart [16,17,18]. Colonies multiply mainly by budding, and mated queens can be adopted back into natal or neighboring nests, so that propagules consisting of a few queens and workers can establish new nests with minimal mate-finding constraints [2,17]. Once established, colonies attain extremely high worker densities [19], raid the nests of other ants, including those of the red imported fire ant *Solenopsis invicta*, and appropriate their brood and territory [20,21]. Its expansion is further shaped by interactions with other invasive ants, because it now co-occurs with the Argentine ant *Linepithema humile* in southern California [22], and in laboratory confrontations in Hawaii it was consistently dominated by the little fire ant *Wasmannia auropunctata*, itself only recently established in Hawaii [23]. These attributes have allowed *P. megacephala* to keep expanding, and new infestation foci are still being reported, from suburban southern California [22] to heated greenhouses in France [11], and species distribution models indicate that large areas of China, including regions well beyond the currently occupied range, remain climatically suitable [5].

Curiously, for an invader of this importance, the basic biology of *P. megacephala* has received far less attention than its taxonomy, genetics or ecological impacts. Unlike many other pest ants, *P. megacephala* does not sting, and although workers may bite when the nest is disturbed, the bite is not painful, so the species poses no direct threat to human health [24]. This low public profile may partly explain why its spread has attracted less media attention, and its basic biology less research effort, than its ecological impact would warrant. Its systematic position and nomenclature are now well resolved [25,26], its foraging, recruitment and predatory behavior have been described in some detail [27,28,29,30], and the genetic architecture of its unicolonial populations is increasingly understood [16,17,18]. By contrast, the fundamental life-history parameters of the species are known only fragmentarily. The number of larval instars was determined for a Cuban population more than four decades ago [31], and the developmental basis of caste determination has been examined at the level of wing imaginal discs [32], but no modern study has described the morphology of the immature stages with the resolution that is now standard in ant larval research [33,34], nor has any study quantified stage-specific developmental schedules, adult longevity, or the seasonal dynamics of caste production in this species, and even its pathogens have only just begun to be characterized, with the first five RNA viruses of the species described in 2025 [35]. This gap matters because life-history traits are the mechanistic link between a species’ environment and its demographic performance, because developmental rate constrains how quickly incipient colonies grow, caste regulation governs how colonies allocate resources between growth and reproduction, and reproductive strategy determines propagule pressure and the probability of establishment in new sites.

Here we provide the first integrated account of the life history of *P. megacephala* based on laboratory rearing and field observations of a population established in subtropical China. Specifically, we describe the external morphology of all developmental stages, including the ultrastructure of larval setae; quantify egg viability, stage-specific developmental durations and adult longevity; document seasonal patterns of caste production in the field and in the laboratory; and characterize nesting site preferences and reproductive behavior. For one of the world’s most widespread invasive ants, such baseline parameters have remained surprisingly fragmentary, and they provide an essential basis both for understanding the adaptive strategies of the species and for developing integrated management. We then ask how this suite of traits, taken together, may promote the establishment and spread of the species, and what it implies for surveillance and control.

## 2. Materials and Methods

### 2.1. Ant Collection and Maintenance

Colonies of *P. megacephala* were collected on the campus of Guangxi University, Nanning, Guangxi Zhuang Autonomous Region, China (22.84° N, 108.29° E), a humid subtropical locality with hot summers and mild winters, a mean annual temperature of 21.8 °C and a mean annual rainfall of 1453.4 mm [36]. Species identity was confirmed from the morphology of minor and major workers following the key and diagnosis in Sarnat et al. [4]. Entire nest fragments, including queens and brood, were excavated from more than fifteen spatially separated nesting sites between May 2022 and June 2024, together with the substrate in which they occurred, and transferred to artificial nests consisting of glass tubes stoppered with a water reservoir behind a cotton plug (“water-plugged tube nests”). Each colony was a complete social unit containing queens, adults of all castes and brood, and all were polygynous, with two to seven dealate queens observed alive per colony during laboratory maintenance. Colonies were maintained in an incubator at 28 ± 1 °C, 70 ± 5% relative humidity and a photoperiod of 16 h light and 8 h dark, and were fed freshly killed mealworms (*Tenebrio molitor* larvae) and an artificial diet (10% sucrose, 1% agar and 89% distilled water by volume, supplemented with a vitamin complex). Nests were cleaned and food replenished at regular intervals. Water-plugged glass tube nests are a standard laboratory setup for rearing ants and have previously been used for colonies of this species [37], and the artificial diet was adapted from the formulation of Bhatkar and Whitcomb [38].

### 2.2. Developmental Observations

Eight colonies were collected and maintained separately for developmental observations. To obtain eggs laid within a 24-h period, all queens from each colony were kept together and transferred to a new water-plugged tube nest (one tube nest per colony). After 24 h, the queens were removed, and approximately 100 workers from the original colony were introduced. The eggs were inspected daily under a stereomicroscope, and we recorded the date of hatching, of each larval molt, of the cessation of feeding (onset of the prepupal stage), of pupation and of adult eclosion. Larval instars were distinguished by head capsule width and general morphology (see Section 3). Once larvae defecated and contracted into the prepupal posture, they were transferred with a soft brush to individual pupation cells (3 mm × 1 mm × 1 mm) cut into plastic blocks, which allowed unambiguous tracking of individuals through pupation. Newly eclosed adults were moved to fresh tube nests for behavioral observation. Because eggs and first- and second-instar larvae cannot be assigned to caste by external morphology (see Section 3), the egg and larval schedule described below was scored on cohorts of non-reproductive brood and is common to brood of all castes. Reproductive brood (gynes and males) was recognized at the third instar, when castes become externally distinguishable, in mature field-collected colonies, and was followed with the same protocol from that stage onwards.

### 2.3. Morphology and Morphometry

Specimens of all developmental stages from the eight colonies were randomly examined, measured and photographed under a Keyence VHX-6000 digital microscope (Keyence, Osaka, Japan). Head capsule width (maximum width across the head), body length and maximum body width were measured on the images. When a photograph did not allow all three measurements on the same individual, the individual was excluded only from the analysis of the missing variable, and all available measurements were retained. For scanning electron microscopy (SEM), specimens were killed and fixed by the hot Dietrich-solution method for 12 h, rinsed three times in 75% ethanol (10 min each), washed six times in 0.1 M phosphate-buffered saline (pH 7.2, 10 min each) and cleaned ultrasonically for 30 s. After dehydration through a graded ethanol series (70, 80, 90 and 100%, 10 min each), specimens were critical-point dried for 1 to 2 h, mounted on stubs with double-sided conductive tape, sputter-coated with gold for 30 s and imaged with an FEI Quattro S field-emission SEM (Thermo Fisher Scientific, Hillsboro, OR, USA).

### 2.4. Adult Longevity Assay

Newly eclosed, still unpigmented (callow) minor workers and soldiers from seven of the eight colonies were used in this assay; all belonged to the same cohort of newly eclosed adults of the same age. Within each colony, minor workers and soldiers were maintained together, and the colonies were kept separately, at 28 °C and 70% RH with ad libitum food and mealworms. Queens were not included in the assay because their lifespan far exceeds the observation period, and males were excluded because they were not provided with mating opportunities. Survival was censused every 14 days, and survivors of each subcaste were recorded separately at each census; to permit accurate counting, the rearing box was placed at 4 °C for 5 min to immobilize the ants. Mean longevity was estimated from the census series as *L* = Σ{[(*d*_i_ + *d*_i+1_)/2] × (*n*_i_ − *n*_i+1_)}/*n*_1_, where *i* indexes the census, *d*_i_ is the number of days since the start of the experiment, and *n*_i_ is the number of survivors at census *i*; this estimator assumes that deaths are distributed uniformly within each 14-day interval. The estimator was applied separately to all non-reproductive adults (*L*_1_), to soldiers (*L*_2_) and to minor workers (*L*_3_).

### 2.5. Nesting Behavior and Reproductive Observations

Between May 2022 and June 2024, nests were located and inspected opportunistically during colony collections on the campus of Guangxi University, and for each nest the substrate, moisture and exposure and the presence or absence of surface spoil were noted. The number of dealate queens per nest was recorded. In addition, the caste composition of brood in field nests and stock colonies was noted during routine inspections throughout the study.

### 2.6. Data Analysis

Normality and homogeneity of variance of the morphometric data were assessed with Shapiro–Wilk and Levene’s tests. Because several groups departed from these assumptions, differences in head capsule width, body length and body width among larval instars and castes were tested with Welch’s analysis of variance followed by Games–Howell post hoc comparisons, which do not assume homogeneity of variance and are robust to deviations from normality. Differences in developmental durations between reproductive and non-reproductive brood were tested by one-way analysis of variance, with significance set at *p* < 0.05. All eight colonies contributed brood to the developmental observations, and the individuals measured were brood sampled randomly from these colonies. Because the offspring of the different queens within each colony were reared together, individual brood items could not be assigned to a particular queen, and because measurements were pooled across colonies, colony could not be included as a factor in the analyses. The data therefore have a nested structure that the present analysis does not fully resolve, and the resulting *p*-values should be interpreted with this limitation in mind. Survival of minor workers and soldiers was summarized with Kaplan–Meier curves, and the two curves were compared with a log-rank test. Because deaths were assigned to the census at which they were detected, survival times are interval-censored, and the Kaplan–Meier estimates approximate the true survival times. This census schedule may slightly bias the absolute longevity values, but because both subcastes were censused on the same schedule, the comparison between them is not affected by this bias. Analyses were run in SPSS v. 19 (IBM Corp., Armonk, NY, USA). Photographs were processed in Adobe Photoshop CC 2019 solely to remove debris and standardize backgrounds; no morphological features were altered.

## 3. Results

### 3.1. The Immature Stages

The life cycle of *P. megacephala* comprises the egg, three larval instars, a prepupal stage, the pupa and the adult.

**Egg** (Figure 1A). Eggs were elliptical, 0.25 ± 0.03 mm long and 0.22 ± 0.09 mm wide (*n* = 30). Freshly laid eggs were milky white and turned translucent with age; the chorion was smooth and glossy with a finely rippled texture and slightly adhesive, so that eggs were frequently found in loose clusters of six to nine.

**Larva, general morphology** (Figure 1B–D). Larvae were apodous, with a well-sclerotized, fully exposed head capsule bearing chewing mouthparts but no eyes; the thorax and abdomen lacked appendages. Two setal types covered the body. Type I setae were short and straight, stout at the base and tapering apically, sometimes forked into a Y-shaped tip (Figure 1C); they occurred sparsely on the venter and evenly elsewhere. Type II setae were long and curled, ending in a bifid, double-hooked (anchor-like) tip (Figure 1D), and were concentrated on the dorsum, which may function to anchor the larva to rough substrates or nest surfaces.

Three instars were recognized based on head capsule width, which differed significantly among instars (Table 1; individual measurements in Table S1). At the third instar, head capsule width did not differ between worker- and soldier-destined larvae, whereas reproductive-destined larvae had slightly but significantly wider head capsules than both non-reproductive groups (Games–Howell, *p* < 0.001). Body size, in contrast, diverged markedly among castes at the third instar (Table 1). First-instar larvae (Figure 1E; body length 0.38 ± 0.06 mm) were translucent, nearly glabrous and slightly C-shaped, with a minute head and simple, bud-like mouthparts. Second-instar larvae (Figure 1F; 0.55 ± 0.09 mm) remained smooth, glossy and translucent, carried only sparse type I setae, and curved into an inverted J; the head was still weakly sclerotized, and the mouthparts remained incompletely developed. The first and second instars could not be assigned to caste by external morphology.

At the third instar, castes became externally distinguishable. Non-reproductive larvae (Figure 2A,B) were fusiform and translucent, densely clothed in both setal types, with a sclerotized and complete chewing mouthpart (labrum, mandibles, maxillae and labium). Soldier-destined larvae (2.45 ± 0.07 mm) were already conspicuously larger than worker-destined larvae (1.38 ± 0.11 mm). Reproductive larvae (Figure 2C) were more than twice as long and almost three times as wide as worker larvae (3.29 ± 0.06 mm long, 1.81 ± 0.13 mm wide), somewhat flattened and fusiform, with a sclerotized head and mature mouthparts. Early third-instar reproductive larvae still carried both setal types densely, but the setal cover thinned progressively as development advanced.

**Prepupa** (Figure 2D–F). Prepupae were slightly smaller than mature larvae, milky white, with a distinctly wrinkled integument, and neither fed nor moved. Non-reproductive prepupae retained their dense setal coat, and soldier prepupae were clearly larger than worker prepupae (Figure 2D,E). Reproductive prepupae, in contrast, were almost glabrous (Figure 2F), with type I setae entirely absent apart from the head and the tip of the abdomen and only a few scattered type II setae remaining on the dorsum.

**Pupa** (Figure 3). Pupae were exarate, with free appendages and the adult body plan already apparent. Fresh pupae were creamy white and translucent with unpigmented compound eyes; with age the cuticle tanned and darkened, the eyes pigmented and the internal structures became visible. Shortly before eclosion, the pupa could perform slight movements. The ontogeny of pigmentation in worker pupae over the first five days is illustrated in Figure 3E.

### 3.2. Adult Castes

Four adult forms were distinguished, namely minor workers, soldiers (major workers), males, and females (virgin gynes and mated, dealate queens).

**Minor worker** (Figure 4). Body length ca. 2.5 mm; cuticle smooth, shining, reddish to dark brown, with sparse standing setae. Mouthparts chewing, labrum reduced, mandibles exposed. Compound eyes well developed, lateral; ocelli absent.

**Soldier (major worker)** (Figure 5). Body length ca. 3.5 mm. Head massive, about 1.3 mm wide, slightly longer than wide, the posterior margin weakly concave; mandibles strongly developed; ocelli absent. Pronotum nearly prismatic in dorsal view. Otherwise as in the minor worker.

**Male** (Figure 6). Body length ca. 5 mm; head small relative to the body; antennae filiform with a short scape; three large dorsal ocelli arranged in a triangle; mesosoma robust, meso- and metathorax each bearing a pair of membranous wings. Males showed positive phototaxis.

**Gyne and queen** (Figure 7). The largest individuals in the colony, 7 to 8 mm long. Mandibles well developed; antennae geniculate with a long scape; three ocelli in a triangle; mesosoma stout, bearing two pairs of membranous wings in virgin gynes, which are shed after mating.

### 3.3. Developmental Schedule

Under the rearing regime (28 ± 1 °C, 70 ± 5% RH), 89.03% of eggs hatched (227 of 255). Hatching within a cohort spread over about three days, and the incubation period lasted 7 to 9 days (mean 7.69 ± 0.63 d; Figure 8 and Table S2). Most larvae (208 of 227) molted to the second instar about two days after hatching (9.54 ± 0.73 d after oviposition), and most of these (194 of 208) molted again to the third instar about seven days after hatching (15.06 ± 0.67 d after oviposition). Non-reproductive third-instar larvae fed for approximately six further days before voiding the gut and entering the prepupal stage (*n* = 93). The prepupal period lasted 2.55 ± 0.19 d, and pupation occurred on average 23.68 ± 2.38 d after oviposition (*n* = 80). The pupal period lasted 5.05 ± 0.45 d (*n* = 52), giving a total egg-to-adult development time of 28.73 ± 2.83 d for non-reproductives. Development was conspicuously asynchronous, and although brood in a cohort had been laid within a 24-h window, the first and last individuals to pupate differed by nine days. Because reproductive brood cannot be distinguished from non-reproductive brood before the third instar, the schedule reported above up to the onset of the prepupal stage applies to all castes, and reproductives accordingly entered the prepupal stage about 21 d after oviposition. Reproductive brood developed significantly more slowly than non-reproductive brood, the difference arising entirely after the larval stage, with prepupal and pupal periods of reproductives lasting 4.16 ± 0.37 d (*n* = 31) and 12.31 ± 0.46 d (*n* = 26), respectively, both significantly longer than in non-reproductives (*p* < 0.05). Pupation and adult eclosion of reproductives occurred on average 25.29 ± 2.74 d and 37.60 ± 3.20 d after oviposition, so that the total egg-to-adult duration of reproductives exceeded that of non-reproductives by about nine days (Figure 8).

### 3.4. Adult Longevity

Survival of newly eclosed non-reproductive adults is summarized in Figure 9. Estimated mean longevity was 29.78 d across all non-reproductives but differed strongly between the two worker subcastes, with soldiers living on average 56 d, more than twice as long as minor workers (26.25 d). Median survival time, taken from the Kaplan–Meier curves, was 70 d for soldiers versus 28 d for minor workers, and the two curves differed significantly (log-rank test, *χ*^2^ = 18.98, df = 1, *p* < 0.001; Figure 9 and Table S3). Soldiers made up 11.86% of the cohort at eclosion, and their share of the surviving population rose steadily as minor workers died off, exceeding 45% by day 56. Longevity also varied considerably among individuals of the same subcaste, and the last minor worker died at day 84, whereas the last soldier survived until day 98.

### 3.5. Nesting Habits and Reproduction

Across field sites, *P. megacephala* consistently nested in moist, shaded situations that offered pre-existing cavities, namely damp rotting logs, the underside of bricks and stones, and accumulations of leaf litter, and indoor activity concentrated in humid rooms such as kitchens and bathrooms. Colonies typically expanded into existing crevices rather than excavating open soil, and the small quantities of spoil they produced never formed surface mounds, in marked contrast with soil-nesting ants such as *Solenopsis invicta*. Nests were accordingly inconspicuous and were usually discovered only after the surface cover was disturbed.

Field and laboratory colonies contained multiple dealate, oviposition queens (polygyny). Oviposition was pulsed rather than continuous. A queen laid a batch of eggs over a short period, then paused until that brood approached the prepupal stage before resuming egg laying, generating a cyclical reproductive rhythm and pronounced overlap of brood of different ages within the colony. At 28 °C with abundant food, one generation (egg to adult worker) required about four weeks, so colonies completed many overlapping generations per year.

Repeated inspections of field nests on campus and of laboratory stock colonies indicated that brood composition varied with season, with only worker-destined brood found through winter and early spring, whereas soldier- and reproductive-destined brood appeared from late spring onwards and sexuals were most frequently encountered between March and May, with males more numerous than gynes.

## 4. Discussion

### 4.1. Larval Instars and Setae as Markers of Caste Ontogeny

Our finding of three larval instars agrees with the only previous instar determination for this species, from a Cuban population [31], and is consistent with the three-instar pattern typical of the genus *Pheidole* and other myrmicines [33,34]. Head capsule width separated the instars without overlap and is therefore a reliable instar marker. Within the third instar, it did not separate worker- from soldier-destined larvae, whereas reproductive-destined larvae had slightly but significantly wider head capsules than non-reproductive larvae. Setal morphology, in contrast, distinguished both instars and castes. First instars were nearly glabrous, second instars bore type I setae, and third instars were dimorphic, because worker- and soldier-destined larvae remained densely setose whereas reproductive larvae progressively shed their setae and became nearly glabrous as prepupae. The two states were unambiguous under the stereomicroscope, because worker- and soldier-destined larvae bore a dense coat of both setal types over the entire dorsum, whereas reproductive larvae lost all type I setae except on the head and the abdominal tip and retained only scattered type II setae on the dorsum. This scoring was qualitative, and formal quantification of setal density per segment would further strengthen the marker. Caste-specific setal patterns have likewise been reported in the leaf-cutting ant *Acromyrmex echinatior* [33] and in the pharaoh ant *Monomorium pharaonis* [34], and caste differences in larval pilosity are also documented in the imported fire ant *Solenopsis invicta* [39] and in *Pheidole pallidula* [40], indicating that larval setal morphology is a practical marker of caste that may apply broadly in myrmicine ants. The early divergence of larval phenotypes further implies that caste determination is already expressed during larval life, consistent with imaginal-disc evidence from this species [32], and it probably involves the nutritional and hormonal pathways that commonly regulate caste in social insects [41]. Such developmental plasticity allows colonies to adjust caste differentiation to changing conditions and may itself be one factor behind the invasive success of *P. megacephala*.

### 4.2. Developmental Schedule and Asynchrony

The developmental schedule we quantified is rapid, with non-reproductives completing egg-to-adult development in under four weeks at 28 °C. Because the egg and larval schedules are shared between castes, the slower development of reproductives accrued entirely in the final stages, in which both the prepupal and the pupal periods were markedly prolonged, adding about nine days to the total egg-to-adult duration. This prolongation presumably reflects the more extensive tissue differentiation and energy accumulation required to build a winged adult [42]. Even the slower reproductive schedule remains fast compared with that of the leaf-cutting ant *Acromyrmex subterraneus*, whose subspecies complete their life cycle in 51 to 77 d [43], and such speed translates directly into fast colony growth. In *P. megacephala*, development is also strongly temperature dependent, because published egg-to-adult schedules for minor workers lengthen from 34 to 38 d at 26–27 °C to an average of 59 d at 24.5–26.7 °C and 66 to 78 d at 20–22 °C, as compiled in [2,44]. Our estimate at 28 °C extends this gradient, and colony growth in the field should therefore slow markedly during the subtropical winter. Development was also strikingly asynchronous, because individuals from eggs laid within 24 h began to diverge after entering the third instar and pupated across a continuous nine-day window. Combined with the pulsed, batch-wise oviposition rhythm, this asynchrony delivers new workers continuously rather than in discrete cohorts and may buffer colonies against environmental fluctuation, adding a further facet to the developmental plasticity noted above.

### 4.3. Seasonal Regulation of Caste Production

Caste production in the study population was seasonally scheduled, with only worker-destined brood through winter and early spring, and all castes, including a spring peak of sexuals, appearing from late spring onwards. Seasonal scheduling of caste production is widespread in *Pheidole* and other ants and is generally linked to photoperiod and temperature [41,45]. The seasonal separation of soldier and reproductive production parallels the developmental trade-off reported in *P. dentata* and *P. morrisi* and may arise because the two castes are similarly large and energetically costly and their developmental pathways are sequentially organized [41,45,46]. The regulatory relationship may nevertheless vary among species and habitats, and the caste investment of an invasive population is probably co-modulated by external environmental cues and intrinsic colony signals. Because our seasonal records are qualitative and come from a single campus population, year-round quantitative sampling across sites is needed to test this hypothesis.

### 4.4. Reproductive Strategies

The social organization and reproductive behavior of *P. megacephala* give it unusual flexibility in colony growth and propagation. The study population was polygynous, with multiple dealate, ovipositing queens coexisting in the same nest and permitting rapid numerical growth. Although colony founding was not observed directly in this study, independent haplometrotic founding by single queens has been reported previously in this species [47]. Polygyny, in combination with such flexibility in colony founding, would maximize dispersal potential and establishment success, especially when populations are small or conditions fluctuate, as in the early stages of an invasion, and probably helps consolidate the ecological advantage of this globally invasive species [2,17]. The batch-wise oviposition we observed parallels an earlier laboratory account in which individual queens deposited eggs in groups on scattered days and laid 97 to 292 eggs over 31 d [44]. In that study, only 38% of eggs developed into larvae because workers and young larvae consumed many of them, whereas most eggs hatched in our queen-separated cohorts, which suggests that egg cannibalism within intact colonies, rather than low intrinsic egg viability, limits the conversion of eggs into brood.

Taken together, the invasion success of *P. megacephala* can be attributed to a coherent suite of biological traits, namely a rapid and plastic developmental schedule, seasonally regulated caste differentiation, cryptic nesting in moist microhabitats and a flexible reproductive system combining polygyny with the reported capacity for single-queen founding. Acting in concert, these traits allow the species to adapt quickly to new environments, exploit resources efficiently and establish persistent populations. By providing the first integrated description of the development and behavior of *P. megacephala*, this study fills a long-standing gap in the life history of a major invasive ant, deepens understanding of its invasion mechanism and identifies seasonal and microhabitat vulnerabilities that surveillance and control can exploit, complementing bait-based approaches already tested against the species [48,49].

## Figures and Tables

**Figure 1:**
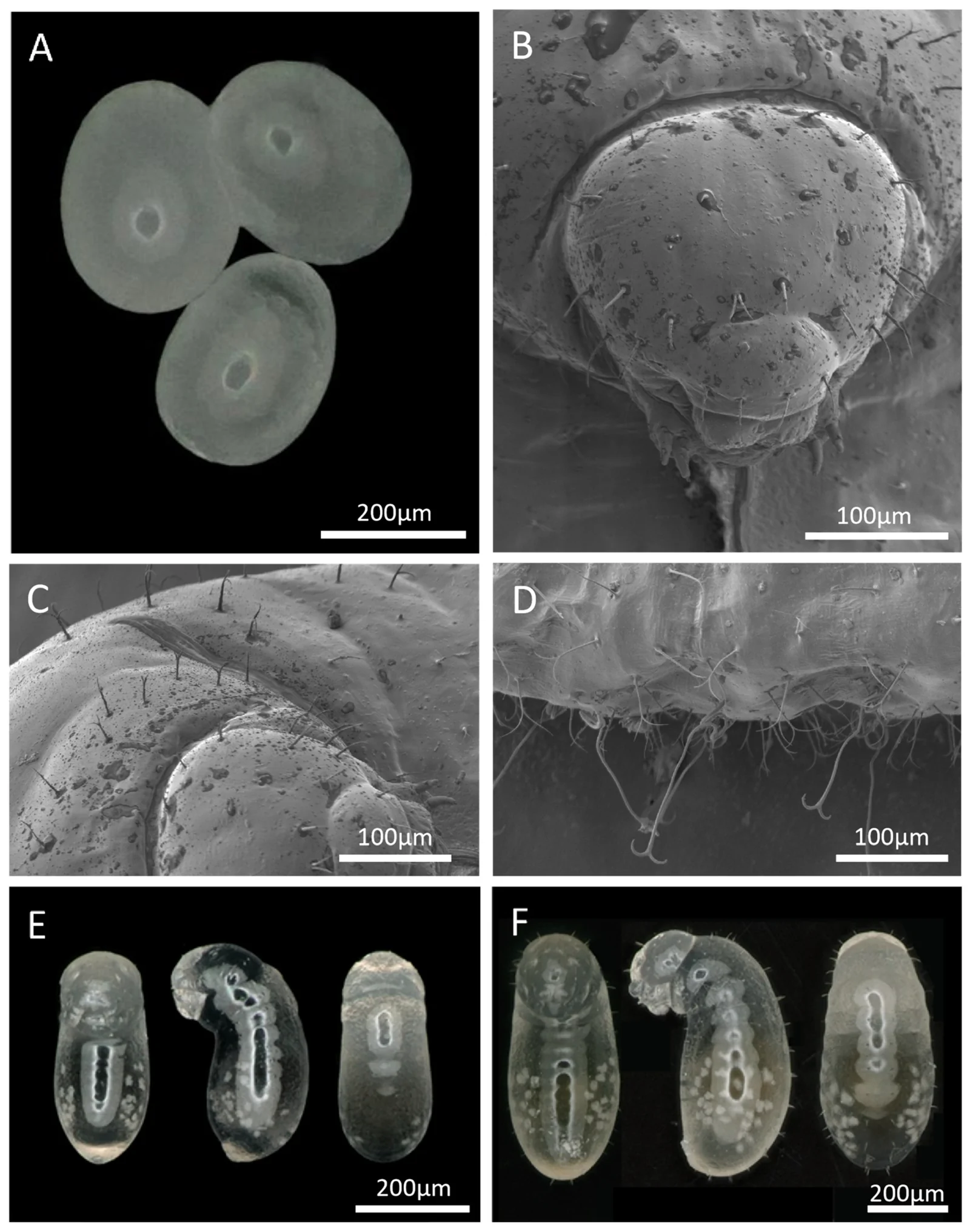
Egg and early-instar larvae of *Pheidole megacephala*. (**A**) Egg; (**B**) larval head capsule with chewing mouthparts; (**C**) type I seta (short, straight, apically Y-forked sometimes); (**D**) type II seta (long, curled, with a bifid anchor-like tip); (**E**) first-instar larva; (**F**) second-instar larva. In (**E**,**F**), views from left to right are ventral, lateral and dorsal.

**Figure 2:**
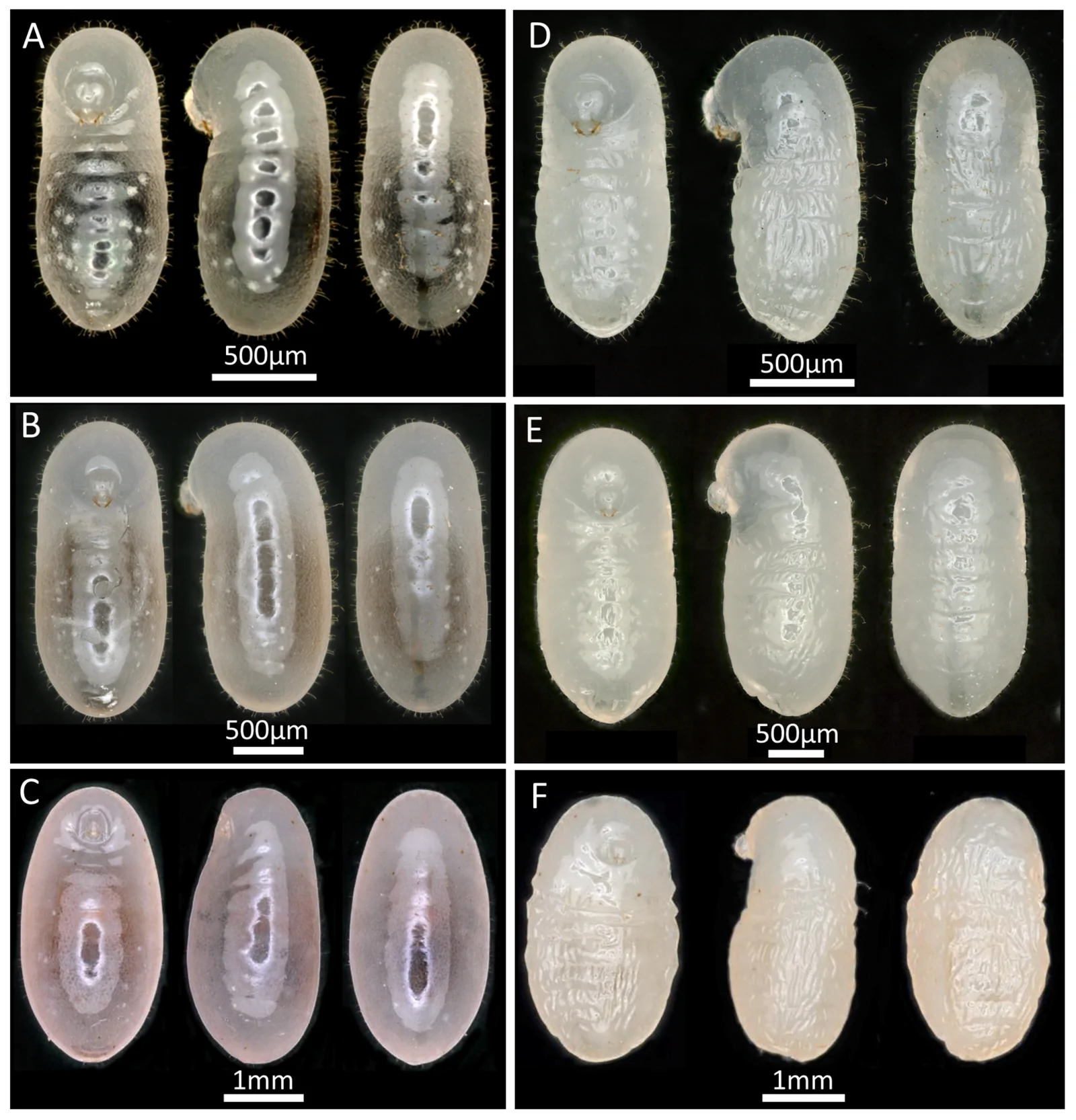
Third-instar larvae and prepupae of *P. megacephala*. (**A**) Worker-destined third-instar larva; (**B**) soldier-destined third-instar larva; (**C**) reproductive third-instar larva; (**D**) worker prepupa; (**E**) soldier prepupa; (**F**) reproductive prepupa; note the nearly glabrous integument. In each panel, views from left to right are ventral, lateral and dorsal.

**Figure 3:**
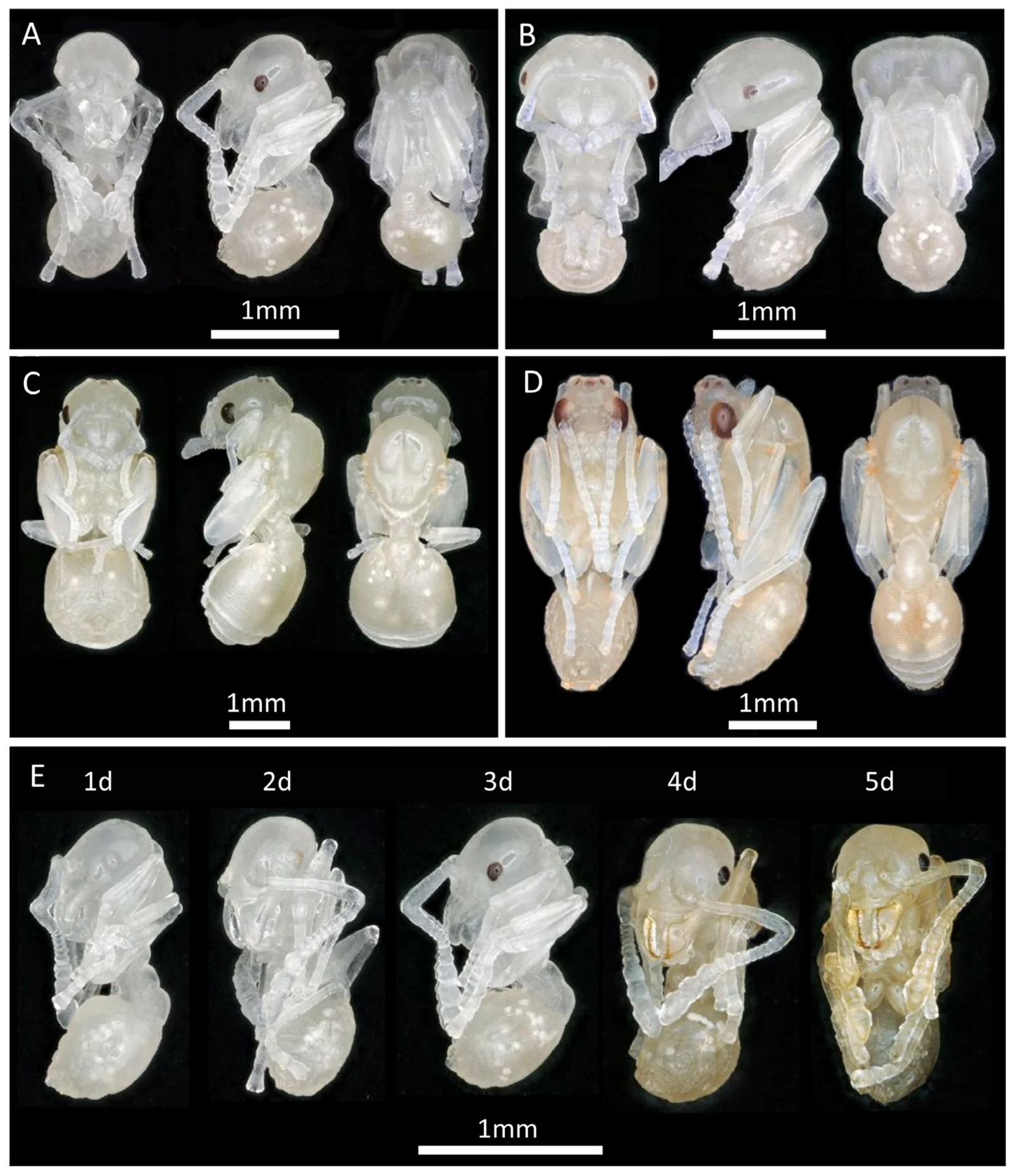
Pupae of the four castes of *P. megacephala* and pigmentation ontogeny of the worker pupa. (**A**) Worker; (**B**) soldier; (**C**) gyne; (**D**) male. For each caste, views from left to right are ventral, lateral and dorsal. (**E**) Worker pupa from day 1 to day 5 after pupation, showing progressive eye pigmentation and cuticular tanning.

**Figure 4:**
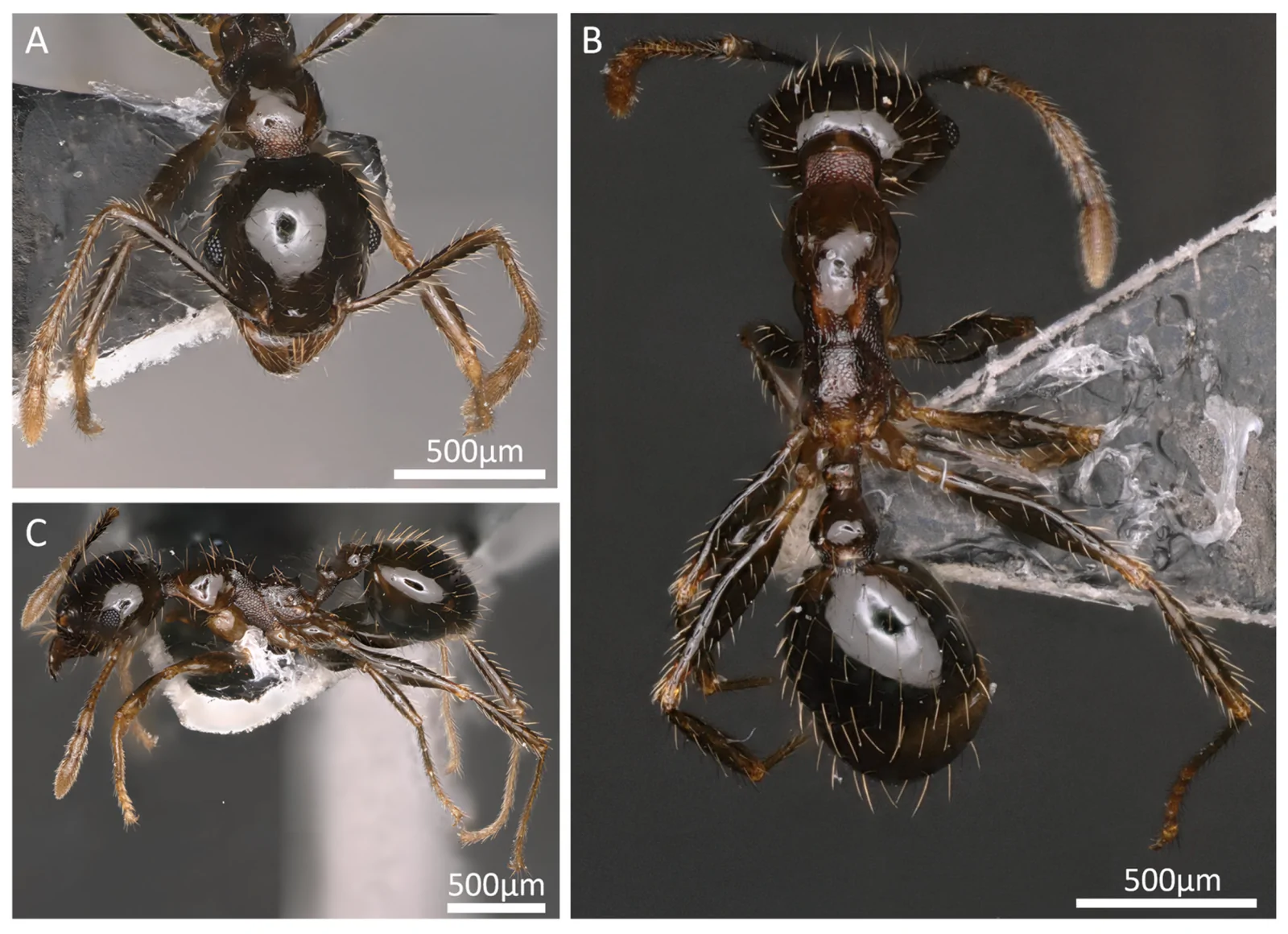
Minor worker of *P. megacephala*. (**A**) Head, frontal view; (**B**) dorsal view; (**C**) lateral view.

**Figure 5:**
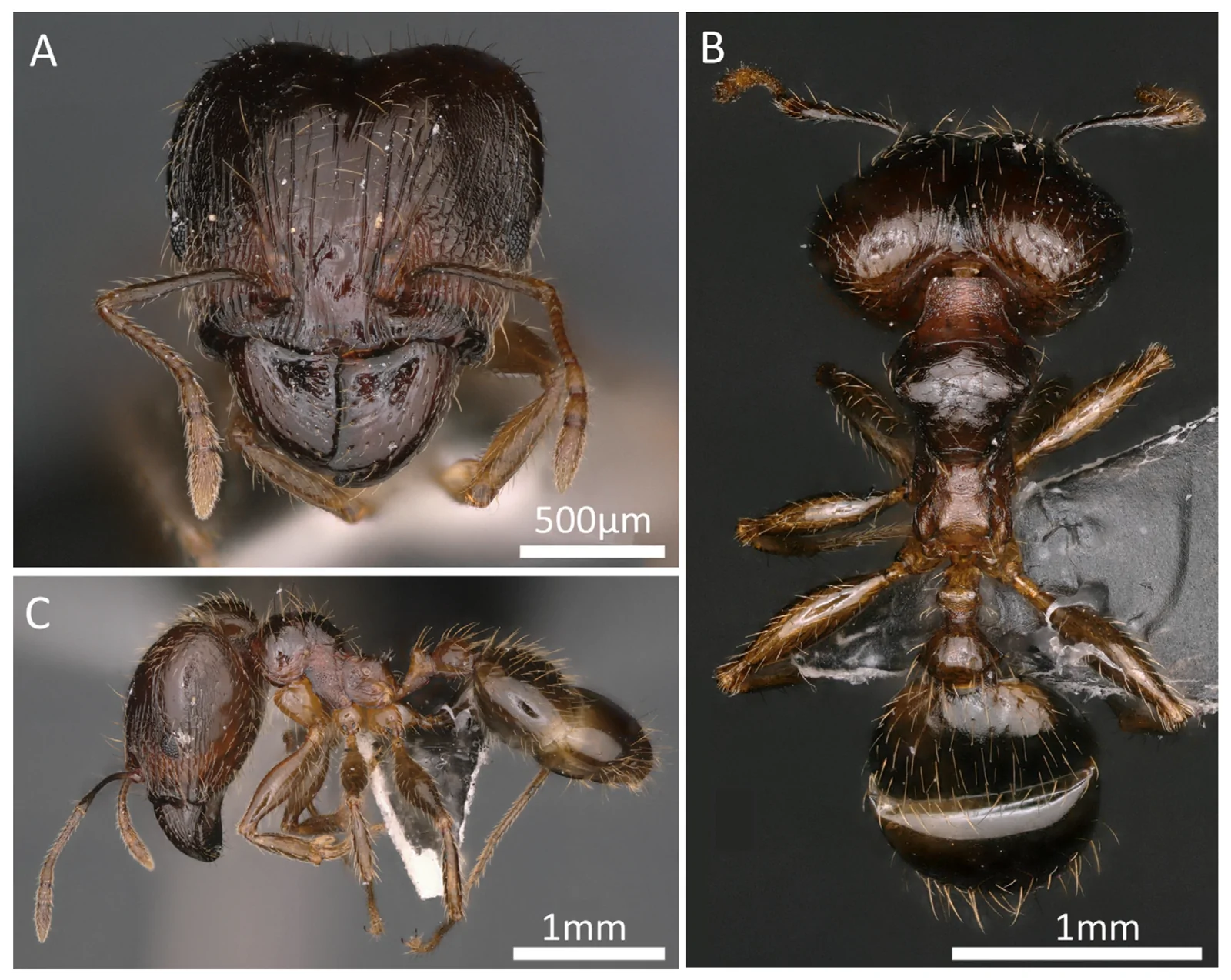
Soldier (major worker) of *P. megacephala*. (**A**) Head, frontal view; (**B**) dorsal view; (**C**) lateral view.

**Figure 6:**
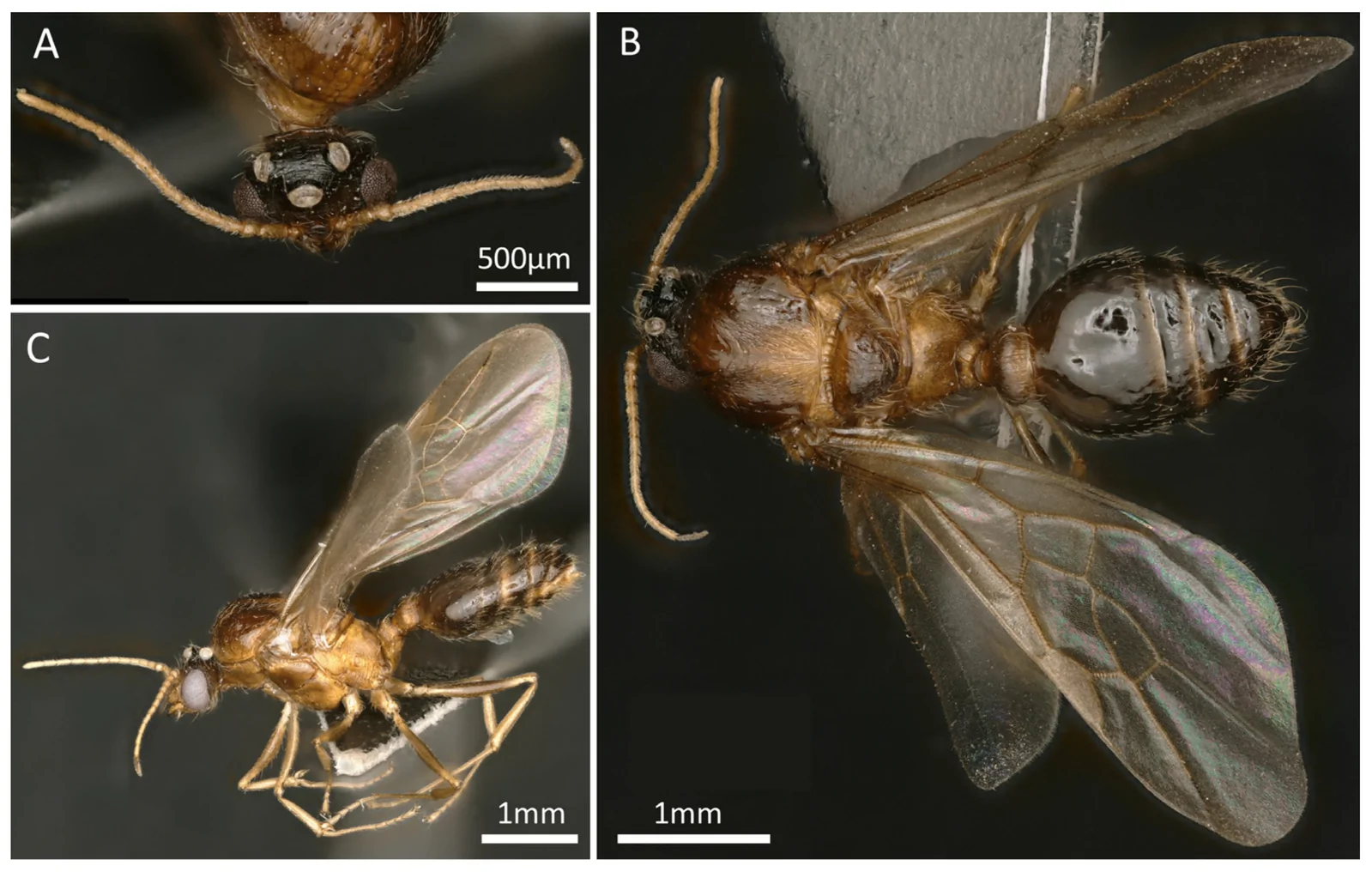
Male of *P. megacephala*. (**A**) Head; (**B**) dorsal view; (**C**) lateral view.

**Figure 7:**
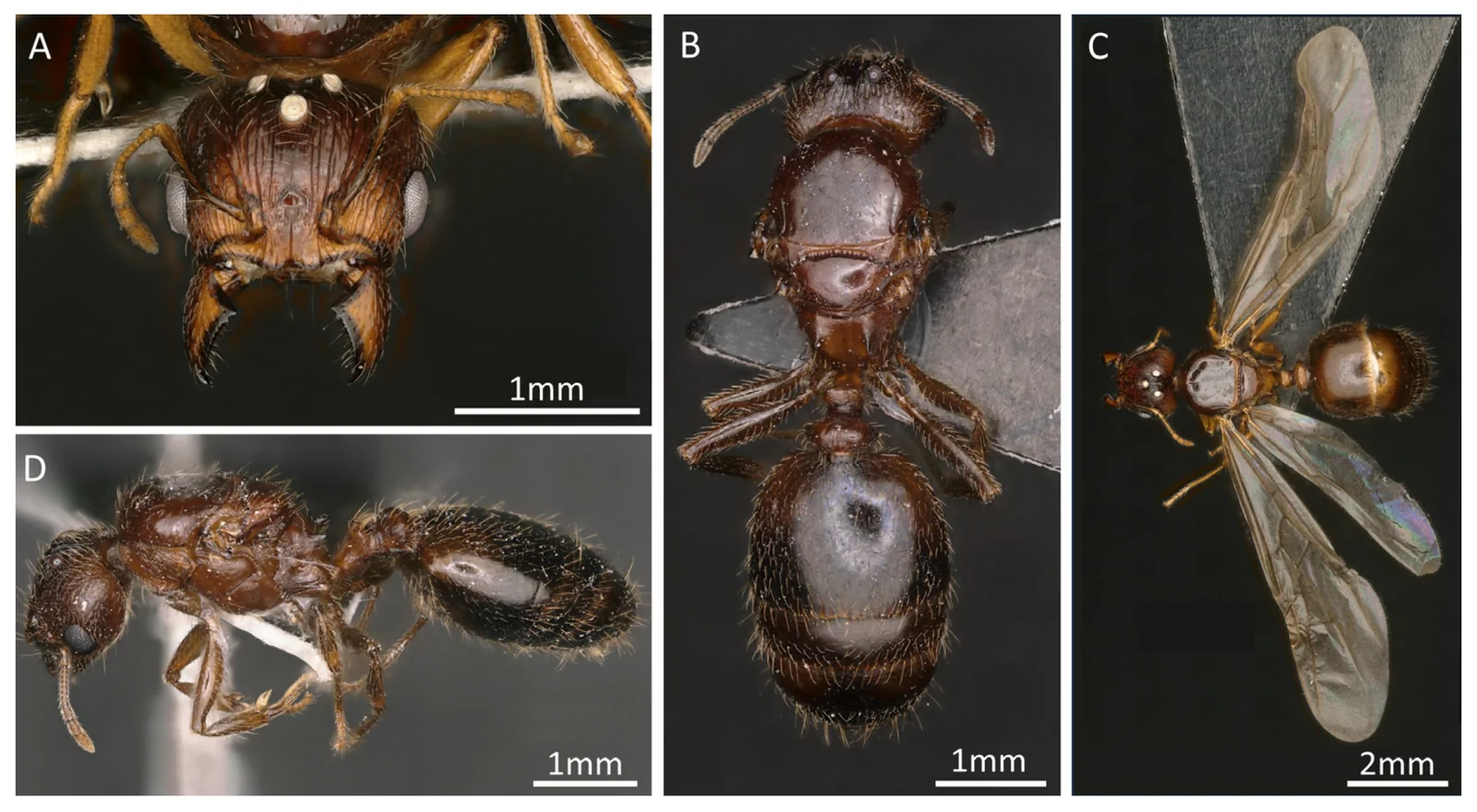
Female sexuals of *P. megacephala*. (**A**) Head of a virgin gyne; (**B**) dorsal view of a dealate queen; (**C**) dorsal view of a winged gyne; (**D**) lateral view of a dealate queen.

**Figure 8:**
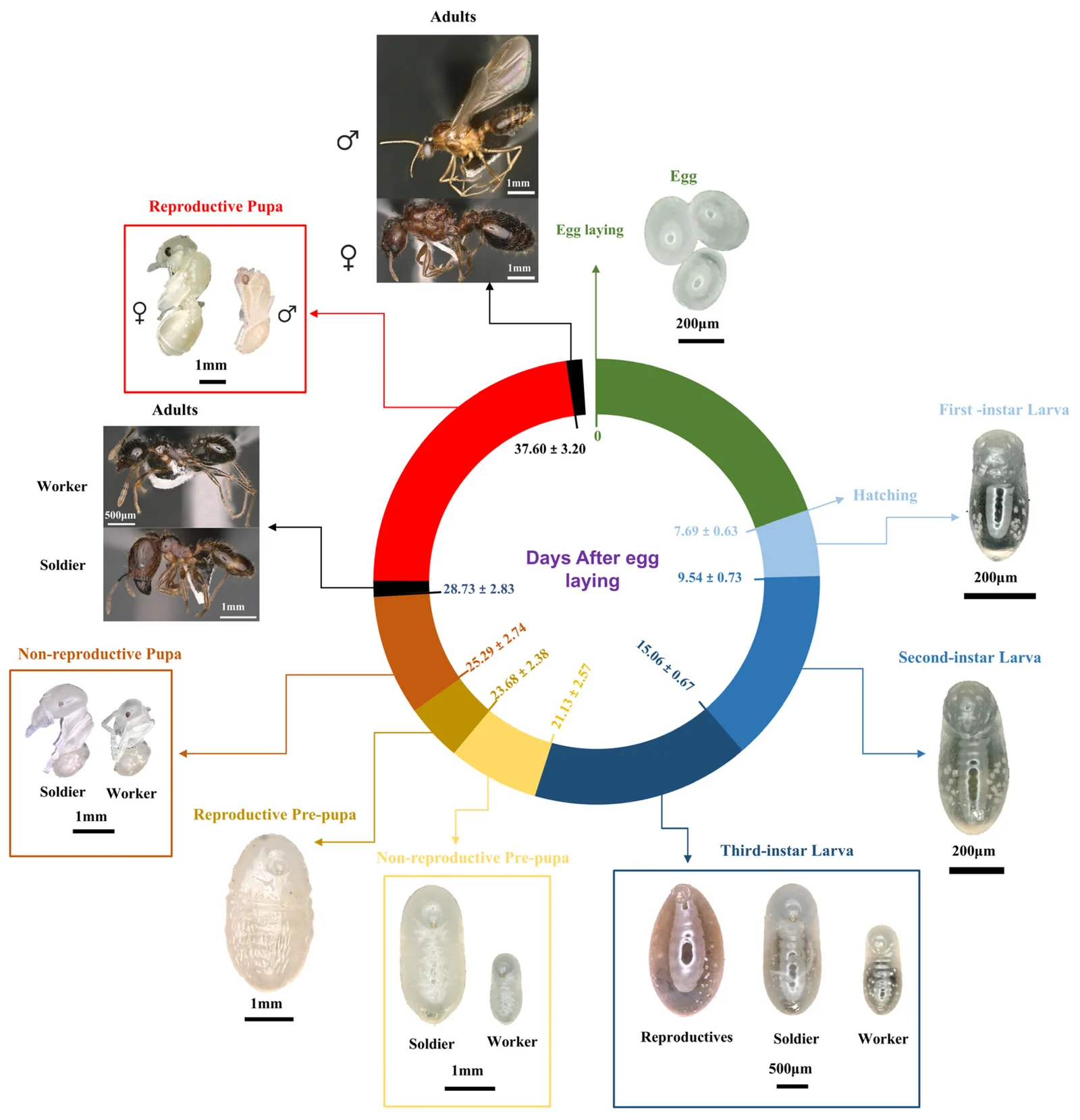
Summary diagram of *P. megacephala* life cycle under laboratory rearing at 28 ± 1 °C and 70 ± 5% relative humidity. The circular timeline is scaled in days after oviposition, with values given as mean ± SD. It begins at egg laying (0 d) and proceeds through hatching (7.69 ± 0.63 d), the molt to the second instar (9.54 ± 0.73 d), the molt to the third instar (15.06 ± 0.67 d) and the onset of the prepupal stage (21.13 ± 2.57 d), a schedule shared by brood of all castes up to that point. The timeline then diverges between the two developmental paths. Non-reproductive brood pupated at 23.68 ± 2.38 d and eclosed as minor workers or soldiers at 28.73 ± 2.83 d, whereas reproductive brood pupated at 25.29 ± 2.74 d and eclosed as gynes or males at 37.60 ± 3.20 d. Representative photographs of each stage are arranged around the timeline. Each color represents a specific period of time. Scale bars are 200 μm for the egg and the first- and second-instar larvae, 500 μm for third-instar larvae and the minor worker, and 1 mm for prepupae, pupae, the soldier and the reproductive adults.

**Figure 9:**
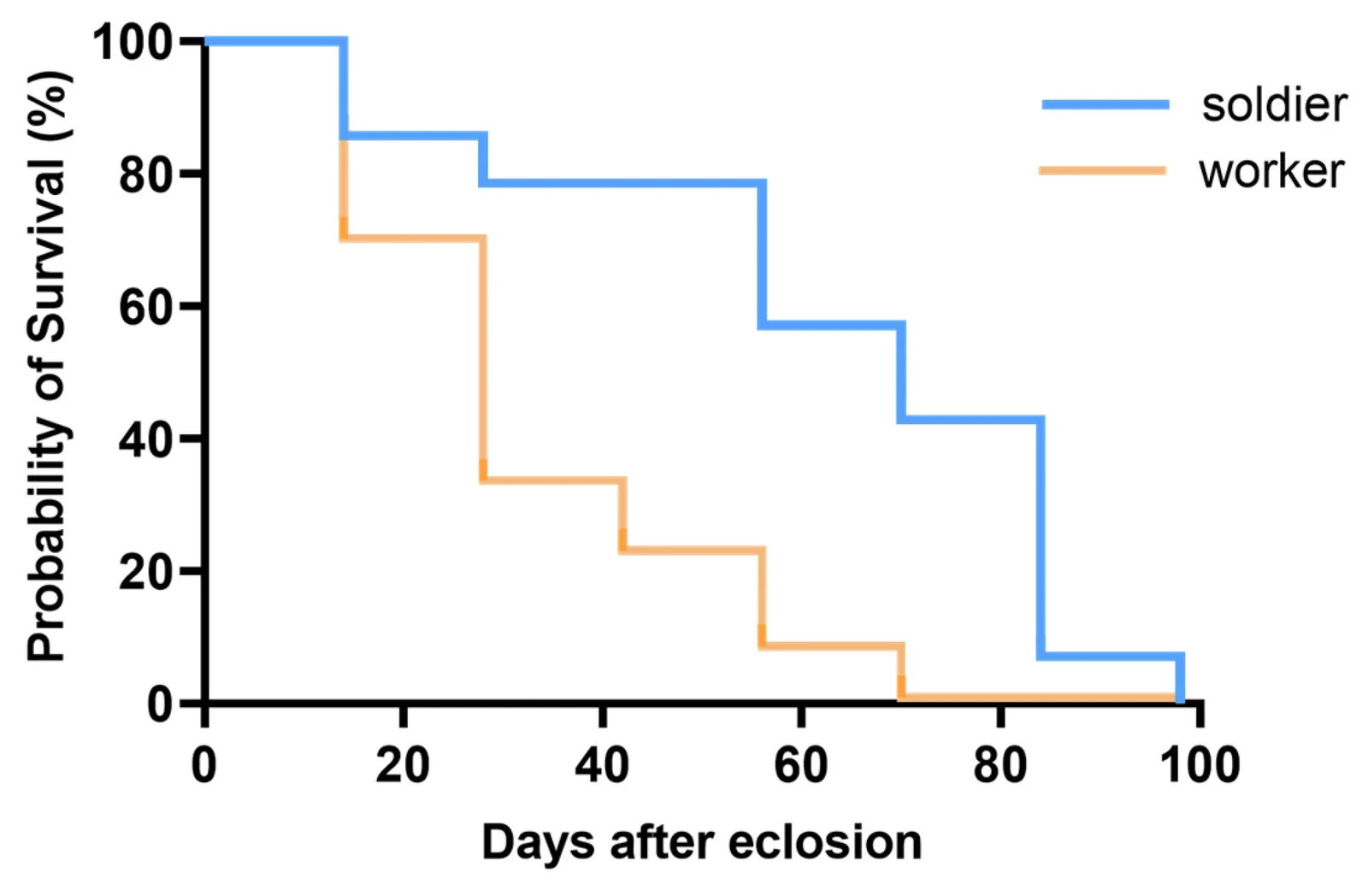
Kaplan–Meier survival curves of newly eclosed minor workers (*n* = 104) and soldiers (*n* = 14) of *P. megacephala* under laboratory conditions (28 °C, 70% RH).

**Table 1:** Morphometric parameters of *Pheidole megacephala* larvae (mean ± SD).

Larval Stage	Caste	Number (*n*=)	Head Capsule Width (mm)	Body Length (mm)	Body Width (mm)
First instar	all	15	0.14 ± 0.01 a	0.38 ± 0.06 a	0.18 ± 0.03 a
Second instar	all	25	0.18 ± 0.01 b	0.55 ± 0.09 b	0.26 ± 0.04 b
Third instar	worker	12	0.24 ± 0.01 c	1.38 ± 0.11 c	0.62 ± 0.03 c
	soldier	8	0.24 ± 0.003 c	2.45 ± 0.07 d	1.09 ± 0.03 d
	reproductive	12 ^†^	0.250 ± 0.002 d	3.29 ± 0.06 e	1.81 ± 0.13 e

Note: Different lowercase letters within a column indicate significant differences among larval stages or catses (Welch’s ANOVA followed by Games–Howell post hoc test, *p* < 0.05). ^†^ Twelve reproductive-destined larvae were examined, of which six were measured completely; because some archived photographs did not allow all three measurements on the same individual, the per-variable sample sizes for this group are *n* = 7 (head capsule width), 11 (body length) and 11 (body width).

## Data Availability

The data supporting the findings of this study are included within the article and its Supplementary Materials. Additional data are available from the corresponding author upon reasonable request.

## Supplementary Materials

The following supporting information can be downloaded at: https://www.mdpi.com/article/10.3390/insects17100992/s1, Table S1: Individual morphometric measurements of *Pheidole megacephala* larvae measured; Table S2: Daily developmental progress of non-reproductive brood of *Pheidole megacephala* reared at 28 °C, expressed as the number of individuals reaching each developmental milestone on each day after oviposition; Table S3: Survival census of newly eclosed non-reproductive adults of *Pheidole megacephala* under laboratory conditions (28 °C, 70 ± 5% RH).
